# Effect of Selected Antioxidants on the In Vitro Aging of Human Fibroblasts

**DOI:** 10.3390/ijms25031529

**Published:** 2024-01-26

**Authors:** Grzegorz Bartosz, Natalia Pieńkowska, Izabela Sadowska-Bartosz

**Affiliations:** Laboratory of Analytical Biochemistry, Institute of Food Technology and Nutrition, College of Natural Sciences, Rzeszow University, Zelwerowicza Street 4, 35-601 Rzeszow, Poland; gbartosz@ur.edu.pl (G.B.); natalia.pien@gmail.com (N.P.)

**Keywords:** fibroblast, in vitro aging, antioxidant, 4-hydroxy-TEMPO, Trolox, ergothioneine, p21, mitochondrial membrane potential

## Abstract

The modification of the replicative lifespan (RLS) of fibroblasts is of interest both from a knowledge point of view and for the attenuation of skin aging. The effect of six antioxidants at a concentration of 1 μM on the replicative lifespan of human dermal fibroblasts was studied. The nitroxide 4-hydroxy-TEMPO (TEMPOL), ergothioneine, and Trolox extended the replicative lifespan (RLS) (40 ± 1 population doublings (PD)) by 7 ± 2, 4 ± 1, and 3 ± 1 PD and lowered the expression of p21 at late passages. Coumaric acid, curcumin and resveratrol did not affect the RLS . The level of reactive oxygen species (ROS) was decreased or not affected by the antioxidants although TEMPOL and coumaric acid decreased the level of glutathione. Only ergothioneine and resveratrol decreased the level of protein carbonylation. The antioxidants that could prolong the RLS elevated the mitochondrial membrane potential. Protecting the activity of mitochondria seems to be important for maintaining the replicative capacity of fibroblasts.

## 1. Introduction

Fibroblasts were the first cells cultured in vitro and are currently the most broadly cultured cells. The aging of fibroblasts both in vitro and in vivo has been extensively studied to understand the mechanisms of aging of mammalian cells. Moreover, fibroblasts are one of the most important cellular components of the skin derma. During the aging of an organism, skin fibroblasts undergo substantial changes in their morphology, functional activity, and proliferative potential, and changes in their functions contribute significantly to the aging of the skin [1,2,3]. Therefore, in vivo fibroblast aging, modeled to some extent by their aging in vitro, is of considerable interest from a cosmetological point of view (amelioration of skin aging).

Two complementary approaches have been employed to limit the deleterious effects of cell aging on the organism: inhibition and delaying of cell aging [4,5,6] and selective destruction of senescent cells to prevent their accumulation with aging (the senolytic approach) [7,8,9].

The reasons for the limitation of the RLS of fibroblasts in vitro are still not obvious. This limitation is mostly ascribed to telomere shortening. The length of telomeric DNA in human fibroblasts decreases as a function of serial passages during aging in vitro. A strong correlation between the replicative capacity of fibroblasts and initial telomere length was found [10,11]. The rate of telomere loss during fibroblast aging in vitro was demonstrated to differ between donors, being much higher for fibroblasts from adult donors than for fibroblasts from infant donors [12]. Apart from cell divisions, oxidative stress may contribute to telomere shortening and single-stranded sites in telomeres were found to increase in non-proliferating fibroblasts [13]. An exponential correlation between cellular oxidative stress levels and telomere shortening rates, independent of donor species and cell strain, was reported. This correlation suggests that oxidative stress-mediated telomere DNA damage is an important determinant of telomere shortening [14]. Given these findings, the application of antioxidants can be expected to delay the in vitro senescence of the fibroblasts.

Fibroblasts have been the object of numerous studies concerning the effects of antioxidants on replicative lifespan (RLS), which was used as the ultimate criterion of in vitro aging of these cells and other symptoms of aging [15]. Early experiments demonstrated a dramatic increase in the RLS of fibroblasts under hypoxia (by 142–157%) [16] and upon the addition of vitamin E to the culture medium (by 68–77%) [17]. However, it was not possible to reproduce the latter results [18] and later studies reported much smaller (if any) effects of exogenous antioxidants. Ascorbic acid [19], ascorbic acid phosphoric ester [20], resveratrol [21], quercetin caprylate [22], cyanidin [23], malvidin [24], 18α-glycyrrhetinic acid [25], carnosine [26], nicotinamide [27,28], α-phenyl-*t*-butyl nitrone [29], several hydroxylamines [30], and methylene blue [31] were reported to prolong the RLS of fibroblasts. The effects of antioxidants were concentration-dependent: in some cases, an increased antioxidant concentration caused a greater increase in RLS [19,30], in other cases, higher antioxidant concentrations were less effective [29]. Some antioxidants were reported not to affect the RLS of fibroblasts [29,30,32,33,34,35] or decreased the RLS [30]. The nitroxides 2,2,6,6-tetramethylpiperidine-1-oxyl (TEMPO), 4-hydroxy-TEMPO (TEMPOL), and 3-carbamoyl-2,2,5,5-tetramethylpyrrolidine-1-oxyl did not affect the RLS of fibroblasts or decreased the RLS, depending on the concentration [30].

Studying the RLS of fibroblasts is a tedious procedure that is not amenable to automatization so no systematic screening of antioxidants for their effect on the RLS of fibroblasts has been performed. However, a search for new antioxidants able to prolong fibroblasts’ RLS seems to be of interest.

**Figure 1:**
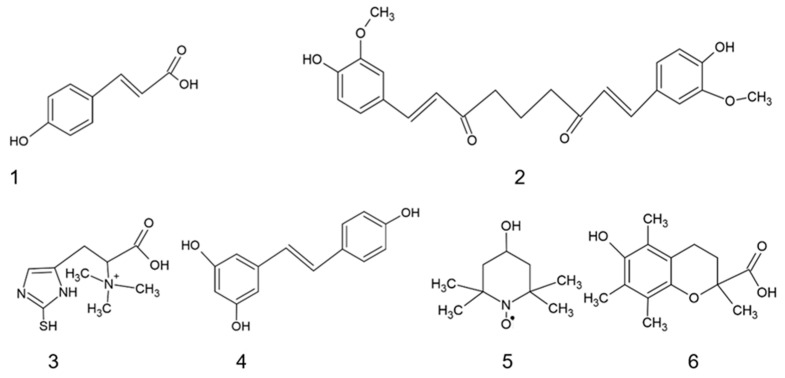
Structures of the antioxidants used: (**1**) *p*-coumaric acid; (**2**) curcumin; (**3**) ergothioneine; (**4**) resveratrol; (**5**) 4-hydroxy-2,2,6,6-tetramethylpiperidin-1-oxyl (TEMPOL); (**6**) Trolox.

In previous experiments, we selected some antioxidants that were able to protect fibroblasts from stress-induced premature aging [36], which prompted us to examine whether they can prolong the RLS of human fibroblasts (Figure 1). Four studied antioxidants (**1**–**4** in Figure 1) are natural compounds, polyphenols (**1**, **2**, **4**), and an important biological thiol (**3**); the two remaining antioxidants (**5**, **6**) are synthetic compounds: a nitroxide stable radical (**5**) and a synthetic vitamin E derivative (**6**). Two of these antioxidants (resveratrol and TEMPOL) were studied previously in this respect with variable results while the effects of the other antioxidants on the RLS of fibroblasts, to the best of our knowledge, have not been studied.

This study was aimed at testing the effects of the above-mentioned antioxidants (*p*-coumaric acid, curcumin, ergothioneine, resveratrol, TEMPOL, and Trolox) on the RLS of human dermal fibroblasts and to obtain insights into the mechanisms of their action by examining their effects on the level of reactive oxygen species, protein carbonylation as a measure of protein post-translational modifications, the level of glutathione, and the mitochondrial membrane potential.

## 2. Results

**Figure 2:**
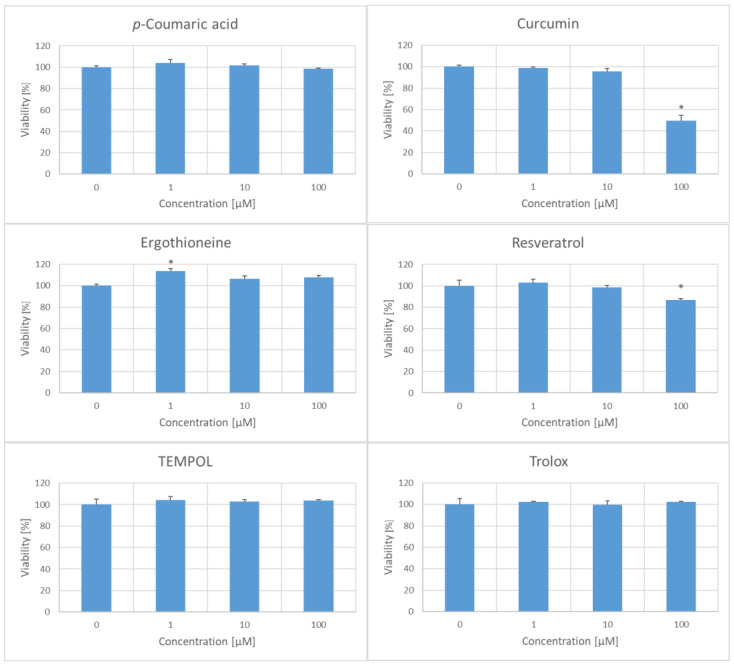
Effect of the viability of H8F2p25LM fibroblasts after 14 h incubation with antioxidants. * *p* < 0.05.

In preliminary experiments, the toxicity of the six chosen antioxidants to the fibroblasts was evaluated. At concentrations of 1 and 10 μM, all tested antioxidants did not impair cell viability, with 1 µM ergothioneine even increasing viability. However, at a concentration of 100 µM, curcumin and resveratrol decreased the viability of fibroblasts (Figure 2). In further experiments, the antioxidants were used at a concentration of 1 µM.

**Figure 3:**
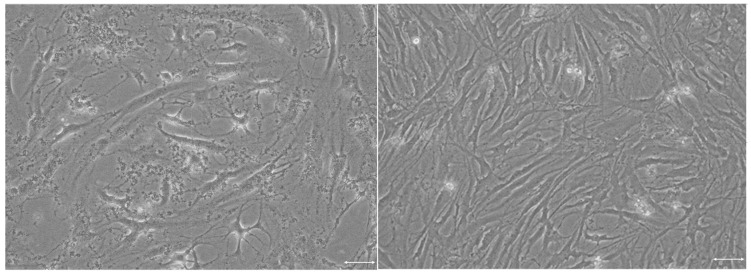
Phase contrast pictures of young (passage 6, **left**) and old (passage 28, **right**) H8F2p25LM fibroblasts. Scale bar: 1 μm.

The aging of the fibroblasts was accompanied by characteristic morphological changes, including an increase in size and shape irregularities (Figure 3), and an increase in the level of the p21 protein.

The replicative lifespan (RLS) of H8F2p25LM fibroblasts corresponded to 40 ± 1 population doublings (PD). Among the antioxidants studied, *p*-coumaric acid, curcumin, and resveratrol did not significantly affect the RLS of the fibroblasts while ergothioneine, TEMPOL, and Trolox significantly extended the RLS of the cells (Table 1).

**Figure 4:**
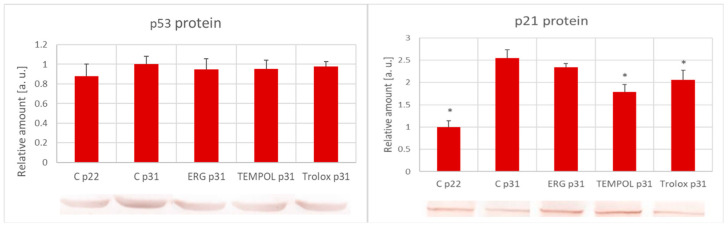
Effect of in vitro aging on the level of p53 and p21 proteins in H8F2p25LM fibroblasts in the absence and presence of antioxidants (1 μM). C, control; ERG, ergothioneine; p22 and p31, passages 22 and 31, respectively. * *p* < 0.05.

No significant increase in the level of p53 protein was found accompanying fibroblast aging in vitro (between passages 22 and 31); the antioxidants prolonging the RLS of the cells did not affect the level of this protein. The level of the p21 protein increased significantly from passage 22 to passage 31; this increase was significantly attenuated by treatment with TEMPOL and Trolox (Figure 4).

**Figure 5:**
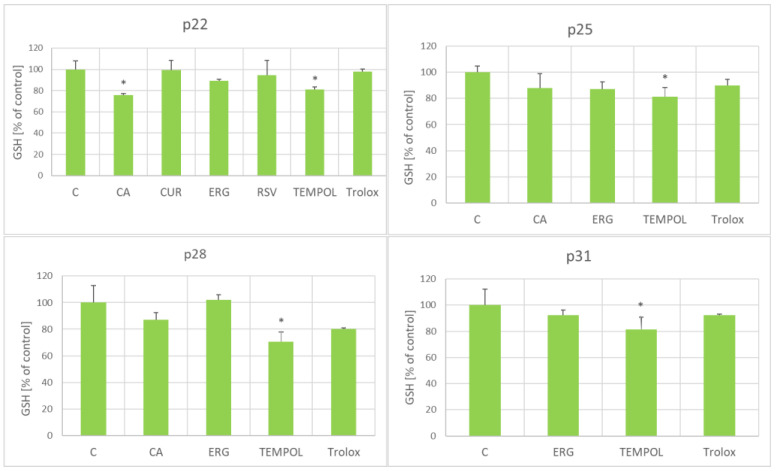
Effect of antioxidants (1 μM) on the glutathione level in aging H8F2p25LM fibroblasts at passages 22, 25, 28, and 31. C, control; CA, *p*-coumaric acid; CUR, curcumin; ERG, ergothioneine; RSV, resveratrol. * *p* < 0.05.

The antioxidants did not affect significantly the glutathione (GSH) level. Only coumaric acid (at passage 22) and TEMPOL (at passages 22, 25, 28, and 31) lowered the GSH level; no antioxidant caused an increase in the GSH level (Figure 5).

**Figure 6:**
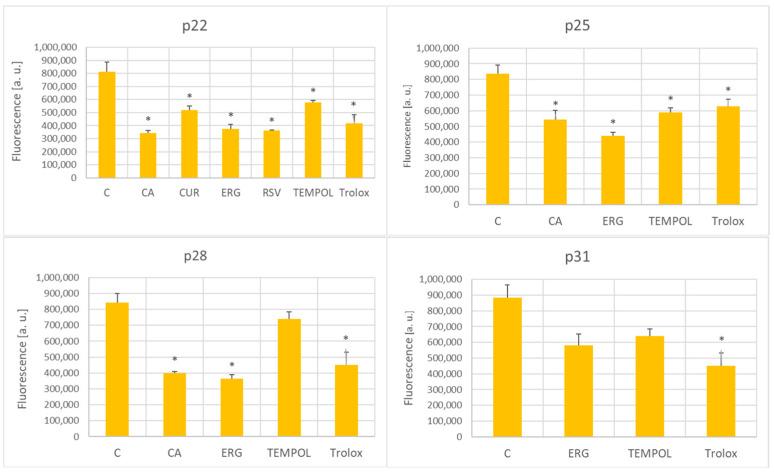
Effect of antioxidants (1 μM) on the ROS levels (estimated using H_2_DCFDA) in aging H8F2p25LM fibroblasts at passages 22, 25, 28, and 31. C, control; CA, *p*-coumaric acid; CUR, curcumin; ERG, ergothioneine; RSV, resveratrol. * *p* < 0.05.

The antioxidants decreased the intracellular level of ROS (estimated using H_2_DCFD) at passages 21 and 25; at passage 28, the ROS level was decreased by *p*-coumaric acid, ergothioneine, and Trolox; at passage 31, this effect was induced only by Trolox (Figure 6).

**Figure 7:**
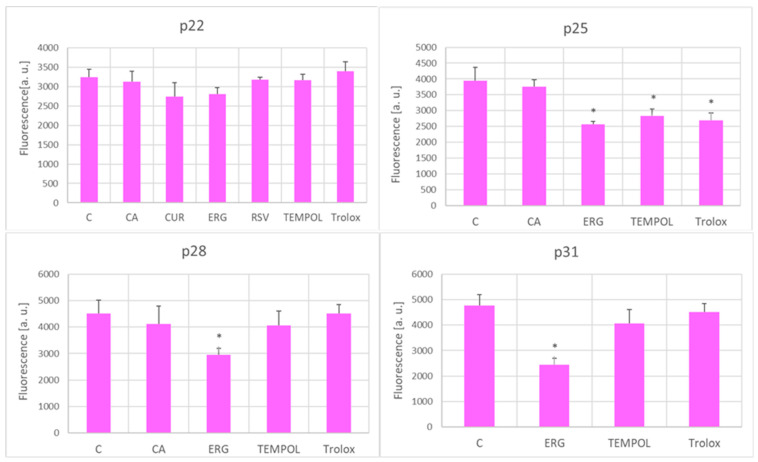
Effect of antioxidants (1 μM) on the ROS level estimated with DHE in aging H8F2p25LM fibroblasts at passages 22, 25, 28, and 31. C, control; CA, *p*-coumaric acid; CUR, curcumin; ERG, ergothioneine; RSV, resveratrol. * *p* < 0.05.

The level of intracellular ROS estimated by DHE, a probe more specific for superoxide, was decreased by ergothioneine at passages 25–31, and also by TEMPOL and Trolox at passage 25 (Figure 7).

**Figure 8:**
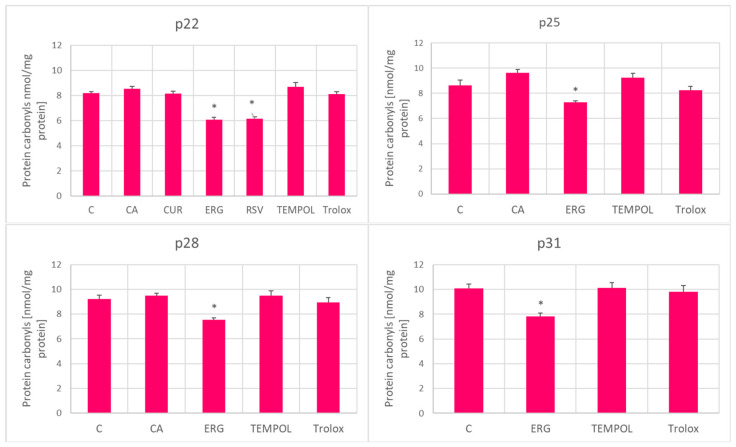
Effect of antioxidants (1 μM) on protein carbonylation in aging H8F2p25LM fibroblasts at passages 22, 25, 28, and 31. C, control; CA, *p*-coumaric acid; CUR, curcumin; ERG, ergothioneine; RSV, resveratrol. * *p* < 0.05.

Protein carbonylation was not significantly affected by treatment with the antioxidants except for ergothioneine, which lowered the level of carbonylation at all stages of aging, and resveratrol which inhibited carbonylation at passage 22 (Figure 8).

**Figure 9:**
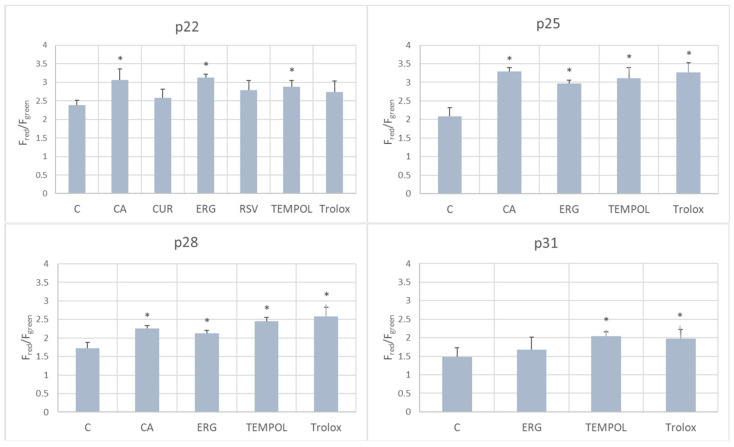
Effect of antioxidants (1 μM) on the mitochondrial membrane potential of aging H8F2p25LM fibroblasts at passages 22, 25, 28, and 31, evaluated using the red to green fluorescence ratio of the JC-1 probe. C, control; CA, *p*-coumaric acid; CUR, curcumin; ERG, ergothioneine; RSV, resveratrol. * *p* < 0.05.

*p*-Coumaric acid, ergothioneine, and TEMPOL increased the mitochondrial membrane potential of the fibroblasts with respect to control cells at passage 22; coumaric acid, ergothioneine, TEMPOL and Trolox increased it at passages 25 and 28; TEMPOL and Trolox elevated the mitochondrial membrane potential at passage p31 (Figure 9).

## 3. Discussion

### 3.1. The Concentration of 1 μM in In Vitro Cell Culture Is Not Low

In the present study, the effect of six chosen antioxidants on the RLS of human fibroblasts in vitro was examined. The antioxidants were applied at a concentration of 1 μM. This concentration may appear low but, as pointed out by Doskey et al. [37], when comparing the effect of a substance on cells, the number of molecules or the number of moles of a substance per cell is more relevant than the concentration of the substance to which the cells are exposed. Let us assume a fibroblast volume of 1900 fL (at a population doubling of 19) [38] and a mean number of cells of 2 × 10^6^ per flask (initially 10^6^ cells were seeded but they replicated during the experiment), and a medium volume of 20 mL in a T75 flask. If an antioxidant is present in the medium at a concentration of 1 μM (10^−6^ mol/L), there are 20 nmoles (20 × 10^−9^ mol) of the antioxidant in the flask. Thus, 20 nmoles of the antioxidant can interact with 2 × 10^6^ cells, i.e., we have a ratio of 10 fmoles (10 × 10^−15^ mol) of the antioxidant per cell with a volume of 1900 fL (1900 × 10^−15^ L). To have the same antioxidant/cell ratio in a body (assuming an equal distribution of the antioxidant in the body), we would have the antioxidant concentration of 1/(1900 × 10^−15^ L) × (10 × 10^−15^ mol) = 5.3 × 10^−3^ mol of the antioxidant per L, i.e., a concentration of 5.3 mM. Such a concentration is impossible to achieve in the body although it can be achieved locally, e.g., by topical application of an antioxidant. Thus, only antioxidant concentrations that are low in absolute terms in in vitro experiments can be physiologically relevant, due to the large excess of the volume of the medium over the cell volume in vitro ( the volume of the medium to the cell volume in vitro (in our experiments 20 mL to 2 × 10^6^ × 1900 fL = ca. 5300-fold higher medium volume than the cell volume).

### 3.2. Three Antioxidants Prolonged the Replicative Lifespan of Human Fibroblasts

The effect of the six chosen antioxidants on the in vitro RLS of H8F2p25LM fibroblasts was studied. *p*-Coumaric acid is the most commonly available form of coumaric acid, a hydroxy derivative of cinnamic acid. It is one of the most significant antioxidants and anti-inflammatory ingredients found in fruits, vegetables, and cereals [39,40] and was reported to inhibit the in vitro aging of rat chondrocytes [41] and nucleus pulposus cells [42]. In *Caenorhabditis elegans*, *p*-coumaric acid decreased the ROS level and increased the stress resistance against oxidative and osmosis stresses; however, it did not influence the lifespan [43]. Curcumin (diferuloylmethane) is a main bioactive polyphenolic compound extracted from the *Curcuma longa* (turmeric) rhizomes and possesses several biological activities, such as antioxidative, anti-inflammatory, anticancer, chemopreventive, and anti-neurodegenerative properties [44]. It was reported to prolong the mean lifespan of model organisms including yeast and *Drosophila melanogaster* [45,46,47,48]. Ergothioneine is a thiol derivative of histidine, with a sulfur atom on the imidazole ring, and has potent antioxidant activity; it is synthesized by a variety of microbes, especially fungi and actinobacteria [49,50], and has been suggested to have multiple anti-aging actions [51] and may prevent age-related diseases [52] and oxidative-stress induced aging of neuronal cells [53]. Resveratrol is a major phytoalexin that was originally isolated from the roots of the oriental medicinal plant *Polygonum cuspidatum*; it is also present in grapes, red wine, peanuts, and blueberries and has antioxidant activity and various biological actions [54]. Resveratrol was reported to prolong the lifespan of yeast, *Caenorhabditis elegans*, and *Drosophila melanogaster* [55] and prevent skin photo-aging [56]. TEMPOL is a synthetic stable nitroxide radical with antioxidant properties [57,58] that is able to reduce inflammation and prolong the lifespan of mice [59]. Trolox (6-hydroxy-2,5,7,8-tetramethylchroman-2-carboxylic acid) is a synthetic vitamin E analog [60] that is used as a standard in antioxidant assays and was found to inhibit the aging of mesenchymal stem cells [61].

Three of the tested antioxidants (*p*-coumaric acid, curcumin, and resveratrol) did not significantly affect the RLS of the fibroblasts while TEMPOL, ergothioneine, and Trolox increased the RLS of the fibroblasts.

The results for resveratrol are consistent with the data of other authors who reported a small increase in RLS [21] or no effect [62] of this compound on the RLS of fibroblasts. TEMPOL was previously found to not affect the RLS of IMR-90 fibroblasts at a concentration of 25 μM and to decrease their lifespan at a concentration of 100 μM [30]. Apparently, there is a therapeutic concentration window for an antioxidant with a maximum positive effect and no or even adverse effects outside the concentration window [4]. The concentration of 100 μM is still far from the cytotoxic concentrations of TEMPOL reported by other authors. The IC_50_ value of TEMPOL, albeit for different cells (HaCaT keratinocytes), was found to be 11.4 mM [63], which is two orders of magnitude higher than that reported to decrease the RLS of fibroblasts [30]. However, there may be considerable differences in the effects of a compound during a 24 h incubation in the estimation of cytotoxicity and the cumulative effects of a compound and its metabolites during a several-month-long experiment aimed at estimating RLSs, with the latter being more sensitive to adverse effects of the exposure. At a concentration of 1 μM, TEMPOL significantly prolonged the fibroblast RLS. Trolox (a vitamin E derivative) and ergothioneine were also able to prolong the RLS of fibroblasts at a concentration of 1 μM.

Apart from RLS, several markers accompany the senescence of fibroblasts. One of the most commonly used is the appearance of acidic β-galactosidase [64]. However, the senescence of H8F2p 25 LM fibroblasts is not accompanied by the expression of acidic β-galactosidase [36]. There are conflicting reports on the effect of fibroblast aging on the level of p53 protein in vitro, with both an increase and lack of change being found [65,66,67,68]. We revealed no significant changes in the p53 level between passages 22 and 31 and no significant effect of the antioxidants. The level of p21 protein is an established marker of fibroblast aging [68,69]. A significant increase in the level of p21 protein was noted during the progression from passage 22 to passage 31. The attenuation of this increase by TEMPOL and Trolox (Figure 4) confirmed the inhibition of cellular senescence by these antioxidants. TEMPOL has been demonstrated to inhibit UVA-induced DNA strand breaks [70]. This finding, together with the inhibition of fibroblast senescence by this compound, suggests the suitability of TEMPOL for application in skin cosmetics.

### 3.3. Antioxidants Attenuated Oxidative Stress and Elevated the Mitochondrial Membrane Potential

The tested antioxidants, except for p-coumaric acid, did not affect the level of glutathione in the fibroblasts (Figure 5). Nitroxides, including TEMPOL, are known to react slowly with glutathione, especially in the presence of oxidants [71]; this reaction may be responsible for the decrease in the glutathione level. Surprisingly, the lower level of glutathione was induced by an antioxidant that did not affect the RLS of the fibroblasts (coumaric acid) and by an antioxidant that increased the RLS (TEMPOL).

All the tested antioxidants, at some stage of the in vitro aging process, decreased the level of ROS (estimated with H_2_DCFDA), although at later passages, this effect was limited to only some of the antioxidants, and only to Trolox at passage 31 (Figure 6). The ROS level estimated with DHE under the applied conditions was claimed to be more specific for superoxide [72]; only the antioxidants that prolonged the RLS decreased the level of DHE-detectable ROS at p25 but later on, this effect was observed only for ergothioneine (Figure 7).

Only ergothioneine and resveratrol decreased the level of protein carbonylation (Figure 8), and thus, this does not seem to be a good predictor of the effect of a compound on the RLS of fibroblasts. In a previous study, only a weak positive correlation was found between oxidative protein damage when comparing primary skin fibroblast lines derived from different donors [13].

None of the applied antioxidants decreased the mitochondrial membrane potential. Most of them and even all of them at passages 25 and 28 energized the mitochondria with respect to control cells (Figure 9). Ergothioneine was reported to reduce hydrogen peroxide production by mitochondria [73]. Growing evidence suggests that ergothioneine is present in mitochondria [50,51]. TEMPOL was found to ameliorate the mitochondrial dysfunction caused by oxidative stress [74,75]. Trolox was reported to restore the mitochondrial membrane potential under oxidative stress conditions [76] and in human complex I deficiency [77]. Thus, the protection of mitochondria seems to be an important property of antioxidants that are able to inhibit the senescence of fibroblasts.

## 4. Materials and Methods

### 4.1. Reagents and Materials

Dulbecco’s Modified Eagle Medium (DMEM) (cat. no. 12430-054, Dulbecco’s Phosphate-Buffered Saline (DPBS) (cat. no. 14040-117) and Protein Assay Kit (cat. no. 23200) were purchased from Thermofisher Scientific (Waltham, MA, USA). Fetal bovine serum (cat. no. 04-001-1A), trypsin–EDTA solution (10×) (cat. no. 03-051-5B), and penicillin–streptomycin solution (cat. no. 03-031-1B) were obtained from Biological Industries (Cromwell, CT, USA). A 0.33% neutral red (NR; CAS 553-24-2) solution (cat. no. N2889), 0.4% trypan blue (CAS 72-57-1) solution (cat. no. T8154), 4-hydroxy-TEMPO (TEMPOL; CAS no. 2226-96-2, cat. no. H8258), L-(+)-ergothioneine (CAS no. 497-30-3, cat. no. E7521), *p*-coumaric acid (CAS 501-98-4, cat. no. C9008), resveratrol (CAS no. 501-36-0, cat. no. R5010), N-ethylmaleimide (NEM) (CAS no. CAS 128-53-0, cat. no. E3876), trichloroacetic acid (TCA) (CAS no. 76-03-9, cat. no. T4885), diethylenetriaminepentaacetic acid (DTPA) (CAS no. 67-43-6, cat. no. D1133), L-ascorbic acid (CAS no. 50-81-7, cat. no. A0278), 2′,7′-dichlorofluorescein diacetate (H_2_DCFDA) (CAS no. 2044-85-1, cat. no. 35845), dihydroethidium (DHE) (CAS no. 104821-25-2, cat. no. 37291), dimethyl sulfoxide (DMSO) (CAS no. 67-68-5; cat. no. D2438), *o*-phtaldialdehyde (OPA) (CAS no. 643-79-8, cat. no. P1378), SIGMAFAST™ Protease Inhibitor Tablets, Trolox (CAS no. 53188-07-1, cat. no. 648471), acrylamide (CAS no. 79-06-1, cat. no. A8887), *N*,*N*′-methylenebis(acrylamide) (CAS no. 110-26-9, cat. no.146072), Tris base (CAS no. 77-86-1, cat. no. 933520, glycine (CAS no. 56-40-6, cat. no. G8898), sodium dodecyl sulfate (CAS no. 151-21-3, cat. no. L3771), hydrogen peroxide (CAS no. 7722-84-1, cat. no. 1.08597), 3,3′-diaminobenzidine (CAS no. 91-95-2)—buffer tablets cat no. 1.02924), bromophenol blue (CAS no. 115-39-9, cat. no. B0126), 2-mercaptoethanol (CAS no. 60-24-2, cat. no. M3148), *N*,*N*,*N*′,*N*′-tetramethyl ethylenediamine (Temed) (CAS no. 110-18-9, cat. no. T9281), ammonium persulfate (CAS no. 7727-54-0, cat. no. A3678), Tween 20 (CAS no. 9005-64-5, cat. no. P9416), bovine serum albumin (CAS no. 9048-46-8, cat. no. A7906), anti-phospho-p21 Cip1 (pThr145) antibody produced in rabbit (cat. no. SAB45044940, anti-p53 antibody produced in rabbit (cat. no. HPA051244), goat anti-rabbit IgG antibody, HRP-conjugate (cat. no. 12-348), and Immobilon-E PVDF Membrane (cat. no. IEVH00005) (cat. no. S8820) were provided by Merck (Poznań, Poland). Ethanol (96%; CAS no. 64-17-5, cat. no. 396420113), glacial acetic acid (CAS no. 64-19-7, cat. no. 568760114), as well as methanol (CAS no. 67-56-1, cat. no. 6219900110) were obtained from Avantor Performance Materials (Gliwice, Poland). Curcumin (CAS no. 67-56-1, cat. no. SC200509) was obtained from Santa Cruz Biotechnology. PhosSTOP Phosphatase Inhibitor Cocktail Tablets (cat. no. PHOSS-RO) were purchased from Roche (Basel, Switzerland). Cell Lysis Buffer (cat.no. FNN0011), JC-1 Mitochondrial Membrane Potential Assay Kit (cat. no. AB113850), and the Protein Carbonyl ELISA Kit (cat. no. AB238536) were purchased from Abcam (Cambridge, UK).

T75 cell culture 75 cm^2^ flasks (cat. no. 156499) were provided by Thermofisher Scientific (Waltham, MA, USA). Transparent 96-well culture plates (cat. no 655180) and black flat-bottom 96-well plates (cat. no. 655209) were obtained from Greiner (Kremsmünster, Austria). Other sterile cell culture materials were provided by Nerbe (Winsen, Germany).

Stock solutions of antioxidants were freshly prepared in DMSO or PBS and filtered through a 0.22 µm filter before each experiment. Absorption and fluorometric measurements were performed using a Spark multimode microplate reader (Tecan Group Ltd., Männedorf, Switzerland).

Human primary fibroblasts (H8F2p 25 LM) isolated from the ear skin of an adult donor were provided by Prof. Jolanta Sroka (Jagiellonian University, Cracow, Poland).

### 4.2. Cell Culture

H8F2p25LM cells were cultured in DMEM supplemented with 1% *v*/*v* penicillin and streptomycin solution and 10% heat-inactivated fetal bovine serum (FBS). The cells were incubated at 37 °C under 5% carbon dioxide and 95% humidity. The cells were passaged at about 85% confluency. For experiments, cells after 16 passages were used. Cell morphology was examined using a Zeiss Primo Vert (Oberkochen, Germany) inverted microscope using phase contrast. Fibroblast viability was estimated using the Trypan Blue exclusion test. The cells were counted using a Thoma hemocytometer (Superior Mrienfeld, Lauda-Königshofen, Germany).

### 4.3. Evaluation of Antioxidant Cytotoxicity

The cells after 16 passages were seeded into a 96-well clear plate at a density of 7.5 × 10^3^ cells/well in 100 µL culture medium and allowed to attach for 24 h at 37 °C. After incubation, the cells were treated with antioxidants at concentrations of 1, 10, and 100 µM. Stock solutions of the antioxidants were prepared in PBS (TEMPOL) or DMSO (other antioxidants). Working solutions of the antioxidants were prepared in the culture medium. The DMSO concentration was adjusted to 0.2% in all samples (including control) except those containing TEMPOL, for which, a control containing no DMSO was used. No significant differences were found between controls containing and not containing 0.2% DMSO in terms of RLS and the other studied parameters. After 24 h of exposure to antioxidants, the medium was removed and replaced with 100 µL of 1% neutral red solution. The plate was incubated for 1 h at 37 °C. Then, the cells were washed with PBS, fixed with 100 µL/well of a solution containing 50% ethanol, 49% H_2_O, and 1% glacial acetic acid, and shaken at 700 rpm at room temperature for 20 min. Absorbance was measured at 540 nm and 620 nm.

### 4.4. Evaluation of the Effect of Antioxidants on Replicative Senescence

Fibroblasts after the 16th passage were seeded into cell culture flasks (T75) at a density of 1 × 10^6^/flask and treated with the antioxidants at 1 µM in the medium. The medium supplemented with antioxidants was exchanged twice a week. Cells were passaged at about 85% confluency (once a week at the beginning of the experiment) and subcultivated in a 1:3 ratio. The cumulative population doubling level at each subcultivation was calculated based on cell counts according to Cristofalo et al. [78]. The experiment was completed when cell division of the cells was observed to stop.

### 4.5. Electrophoresis and Western Blotting

The cells were washed with PBS and lysed with a Cell Lysis Buffer (Abcam) with PhosSTOP Phosphatase Inhibitor Cocktail (Roche) as recommended by the manufacturer. The lysates were frozen at −80 °C until electrophoresis. The lysates (30 μg protein) were separated in 12% SDS-polyacrylamide gel (200 V, 40 min) in a Mini Trans-Blot^®^ BioRad apparatus and transferred onto Immobilon membranes (30 V, 16 h) in the same apparatus according to the instructions of the manufacturer. The Immobilon membranes were washed thrice with TBST (Tris-HCl-buffered saline with 0.1% Tween 20), blocked with 3% bovine serum albumin (1 h, RT), washed 3 times with TBST, and incubated overnight with primary antibodies (1:1000) at 4 °C. After washing with TBTS (5 times), the Immobilon membranes were incubated with HRP-conjugated secondary antibodies (1:1000), washed with TBST (5×), and stained in a 3,3-diaminobenzidine (DAB) solution (one DAB buffer tablet dissolved in 15 mL H_2_O supplemented with 13 μL 30% hydrogen peroxide). The blots were scanned and the band intensity was estimated using ImageJ Version 1.51w software (NIH, Bethesda, MD, USA) (http://rsb.info.nih.gov/ij/, accessed 23 December 2023).

### 4.6. Estimation of the Content of Glutathione

The content of reduced glutathione (GSH) was measured with *ortho*-phtalaldehyde (OPA) according to Senft et al. [79]. The cells were seeded into the wells of a 96-well clear plate (7.5 × 10^3^ cells/well) and allowed to attach for 24 h at 37 °C. Then, the medium was removed, the cells were washed with PBS (150 µL per well), and then 60 µL/well of cold Redox Quenching Buffer (RQB) containing 20 mM HCl, 5% trichloroacetic acid (TCA), 5 mM diethylenetriaminepentaacetic acid, and 10 mM L-ascorbic acid was added. The plate was shaken for 5 min and centrifuged for 5 min at 4000 rpm. Next, the cell lysates were transferred into two 96-well black bottom plates (+NEM and −NEM) in an amount of 25 µL/well. Within the + NEM plate, 4 µL/well of freshly prepared 7.5 mM *N*-ethylmaleimide (NEM) in cold RQB buffer was added. Then, 40 µL/well of 1 M phosphate buffer (pH 7) was added to the wells of both plates and the plates were shaken at 700 rpm for 5 min. Finally, 160 µL/well of cold 0.1 M phosphate buffer (pH 6.8) and 25 µL/well of freshly prepared 0.5% OPA in methanol were added to the wells of both plates. Both plates were incubated at room temperature with constant shaking for 30 min. Fluorescence was measured at 355/430 nm. The protein content of the cell lysates was determined according to the protocol of Lowry et al. [80]. The concentration of reduced glutathione was determined by subtracting the fluorescence of the +NEM plate from the fluorescence of the -NEM plate and calculated with respect to the protein content.

### 4.7. Evaluation of the Level of Reactive Oxygen Species

The level of ROS in fibroblasts treated with antioxidants was estimated with 2′,7′-dichlorofluorescein diacetate (H_2_DCFDA) and dihydroethidium (DHE). The cells were seeded in a 96-well flat clear-bottom black plate at an amount 7.5 × 10^3^/well and allowed to attach at 37 °C for 24 h. Subsequently medium was removed and replaced by a 10 µM solution of H_2_DCFDA or DHE in PBS (100 µL/well). The stock solutions of the probes were prepared in DMSO and diluted with PBS. Fluorescence was measured at 490/529 nm when using H_2_DCFDA and 405/570 nm when using DHE to increase its specificity for the detection of the superoxide reaction product [61] every minute for 2 h at 37 °C. The sum of the fluorescence values measured (“area under the curve”) was assumed as a measure of the level of ROS.

### 4.8. Evaluation of Changes in the Mitochondrial Membrane Potential

To estimate changes in the mitochondrial membrane potential, a JC-1 Mitochondrial Membrane Potential Assay kit (Abnova) was used. Briefly, fibroblasts treated with various antioxidants after 22, 25, 28, and 31 passages (corresponding to about 31, 35, 39 and 43 PD) were seeded into 96-well flat clear-bottom black plates at a density 7.5 × 10^3^/well and allowed to attach for 24 h at 37 °C. Then, 10 µL of JC-1 staining solution was added into the medium of each well and the plate was incubated at 37 °C for 30 min, then centrifuged at 4000 rpm for 5 min in an Eppendorf 5810R centrifuge and the supernatants were gently removed. The wells were washed twice using the buffer included in the kit, with centrifugation to avoid cell loss. The final supernatant was replaced by the buffer provided by the kit manufacturer (100 µL/well) and fluorescence was measured at 535/595 nm (JC-1 aggregates) and 485/535 nm (JC-1 monomers). The results are shown as a ratio of the fluorescence of JC-1 aggregates (red) to the fluorescence of JC-2 monomers (green).

### 4.9. Statistics

The results are presented as the mean and SD from three independent experiments. To estimate the statistical significance of differences, ANOVA was used; differences with *p* < 0.05 were considered statistically significant and denoted with *. The statistical analyses of the data were performed using the STATISTICA software package (version 13.1, Statsoft Inc. 2016, Tulsa, OK, USA).

## 5. Conclusions

The presented results document the prolongation of fibroblast RLSs in vitro by 1 μM nitroxide TEMPOL, Trolox, and ergothioneine. This effect seems to be connected to the ability of these compounds to preserve the functions of mitochondria and lower ROS production. These data contribute to the knowledge of the beneficial effects of antioxidants and strengthens the suggestions for the use of TEMPOL in cosmetics as not only an agent protecting from UV, but also a compound that can retard fibroblast aging. The action of antioxidants depends on the concentration so the concentration dependence of the effects of the these antioxidants should be examined to find the concentration window for their protective effect and optimal concentrations.

## Figures and Tables

**Table 1:** Effect of antioxidants on the replicative lifespan (RLS) of H8F2p25LM fibroblasts (number of population doublings (PD)).

Antioxidant	RLS (PD)
None	40 ± 1
*p*-Coumaric acid	39 ± 1
Curcumin	37 ± 3
Ergothioneine	44 ± 1 *
Resveratrol	38 ± 2
TEMPOL	47 ± 2 *
Trolox	43 ± 1 *

Mean ± SD; * *p* < 0.05.

## Data Availability

Data are available from the corresponding author upon reasonable request.
